# Supplementary material for: Deletion of wfs1 Impairs Oligodendrocyte Precursor Cells Dorsal Distribution and Myelination Through the wfs1-hmgcs1 Axis in Zebrafish
Source: Biology (Basel). 2026 Aug 16;15(16):1402. doi: 10.3390/biology15161402 (PMC13510151; doi:10.3390/biology15161402)
Supplement: Supplementary file 1 [file biology-15-01402-s001.zip › File S1-Transcriptome sequencing data and differentially expressed genes (DEGs) in wfs1-- zebrafish at 5 dpf/2.KEGG/wfs1KOvsWT/GSEA/WT/FERROPTOSIS/FERROPTOSIS(DRE04216).html]

Details for gene set FERROPTOSIS(DRE04216)[GSEA]

|  || Dataset | deg.sample |
| Phenotype | sample.cls |
| Upregulated in class | WT |
| GeneSet | FERROPTOSIS(DRE04216) |
| Enrichment Score (ES) | -0.31595108 |
| Normalized Enrichment Score (NES) | -1.4573245 |
| Nominal p-value | 0.032520324 |
| FDR q-value | 0.57249916 |
| FWER p-Value | 0.81 |
Table: GSEA Results Summary

  

Fig 1: Enrichment plot: FERROPTOSIS(DRE04216)      
 Profile of the Running ES Score & Positions of GeneSet Members on the Rank Ordered List

  

| PROBE | DESCRIPTION (from dataset) | GENE SYMBOL | GENE\_TITLE | RANK IN GENE LIST | RANK METRIC SCORE | RUNNING ES | CORE ENRICHMENT || 1 | 393186\_1 | zgc:56235 |  |  | 453 | 3.118 | 0.0460 | No |
| 2 | 796322 | slc3a2a |  |  | 483 | 3.041 | 0.1038 | No |
| 3 | 450084\_1 | gss |  |  | 836 | 2.334 | 0.1378 | No |
| 4 | 108182827 | LOC108182827 |  |  | 2311 | 1.246 | 0.1155 | No |
| 5 | 565700 | sat1a.1 |  |  | 2458 | 1.187 | 0.1338 | No |
| 6 | 30590\_1 | tp53 |  |  | 2944 | 1.051 | 0.1388 | No |
| 7 | 393622 | acsl4a |  |  | 3304 | 0.972 | 0.1463 | No |
| 8 | 553552 | zgc:109934 |  |  | 4319 | 0.773 | 0.1294 | No |
| 9 | 84702 | cp |  |  | 4415 | 0.758 | 0.1410 | No |
| 10 | 100330105 | map1lc3cl |  |  | 5232 | 0.652 | 0.1280 | No |
| 11 | 447860 | acsl5 |  |  | 5835 | 0.604 | 0.1207 | No |
| 12 | 494477 | tfr1a |  |  | 6320 | 0.555 | 0.1162 | No |
| 13 | 494478 | tfr1b |  |  | 7066 | 0.486 | 0.1022 | No |
| 14 | 326857 | gclc |  |  | 7445 | 0.457 | 0.0991 | No |
| 15 | 678623 | slc11a2 |  |  | 7514 | 0.454 | 0.1057 | No |
| 16 | 447823 | fth1b |  |  | 7921 | 0.429 | 0.1013 | No |
| 17 | 555566\_1 | lpcat3 |  |  | 8121 | 0.417 | 0.1031 | No |
| 18 | 100151597 | slc7a11 |  |  | 9607 | 0.327 | 0.0627 | No |
| 19 | 406466 | map1lc3a |  |  | 9731 | 0.319 | 0.0650 | No |
| 20 | 352928 | gpx4a |  |  | 9915 | 0.309 | 0.0652 | No |
| 21 | 30255 | tfa |  |  | 11374 | 0.223 | 0.0237 | No |
| 22 | 555566 | lpcat3 |  |  | 11406 | 0.222 | 0.0270 | No |
| 23 | 559656 | acsl3b |  |  | 11481 | 0.218 | 0.0289 | No |
| 24 | 393386 | cybb |  |  | 11782 | 0.202 | 0.0234 | No |
| 25 | 399488 | slc3a2b |  |  | 11812 | 0.200 | 0.0263 | No |
| 26 | 566863 | acsl6 |  |  | 12701 | 0.154 | 0.0014 | No |
| 27 | 100151035 | si:ch211-254p10.2 |  |  | 13929 | 0.096 | -0.0353 | No |
| 28 | 567881\_1 | sat1b |  |  | 14419 | 0.073 | -0.0493 | No |
| 29 | 494180 | atg5 |  |  | 14638 | 0.063 | -0.0549 | No |
| 30 | 321844 | pcbp2 |  |  | 14786 | 0.056 | -0.0585 | No |
| 31 | 450084 | gss |  |  | 14972 | 0.048 | -0.0633 | No |
| 32 | 568699 | si:ch73-352p4.8 |  |  | 14993 | 0.047 | -0.0631 | No |
| 33 | 100006523 | zgc:198419 |  |  | 16166 | -0.007 | -0.0998 | No |
| 34 | 494478\_1 | tfr1b |  |  | 16422 | -0.015 | -0.1075 | No |
| 35 | 334561 | acsl4b |  |  | 16648 | -0.022 | -0.1141 | No |
| 36 | 445175 | acsl1b |  |  | 16699 | -0.023 | -0.1153 | No |
| 37 | 333974 | gclm |  |  | 17390 | -0.049 | -0.1360 | No |
| 38 | 406529 | vdac3 |  |  | 17989 | -0.075 | -0.1534 | No |
| 39 | 555308 | acsl1a |  |  | 18027 | -0.077 | -0.1530 | No |
| 40 | 799782 | slc39a14 |  |  | 19147 | -0.129 | -0.1857 | No |
| 41 | 567157 | acsl3a |  |  | 19451 | -0.142 | -0.1925 | No |
| 42 | 569184\_1 | si:dkey-19b23.15 |  |  | 20590 | -0.193 | -0.2245 | No |
| 43 | 406286 | zgc:56095 |  |  | 21686 | -0.251 | -0.2541 | No |
| 44 | 100332846 | acsl2 |  |  | 21878 | -0.261 | -0.2551 | No |
| 45 | 322126 | vdac2 |  |  | 22259 | -0.282 | -0.2616 | No |
| 46 | 58153 | slc40a1 |  |  | 22602 | -0.302 | -0.2665 | No |
| 47 | 394104 | ncoa4 |  |  | 22685 | -0.307 | -0.2632 | No |
| 48 | 58108 | fth1a |  |  | 23202 | -0.341 | -0.2728 | No |
| 49 | 494055\_1 | sat2a |  |  | 23545 | -0.364 | -0.2765 | No |
| 50 | 436827 | sat2b |  |  | 23725 | -0.375 | -0.2749 | No |
| 51 | 555308\_1 | acsl1a |  |  | 24418 | -0.416 | -0.2886 | No |
| 52 | 100333016 | atg7 |  |  | 25106 | -0.454 | -0.3015 | No |
| 53 | 791518 | hmox1a |  |  | 25490 | -0.483 | -0.3042 | No |
| 54 | 790938\_1 | zgc:158423 |  |  | 25805 | -0.511 | -0.3042 | No |
| 55 | 567881 | sat1b |  |  | 26164 | -0.544 | -0.3049 | Yes |
| 56 | 431716 | sat1a.2 |  |  | 26516 | -0.580 | -0.3048 | Yes |
| 57 | 555566\_2 | lpcat3 |  |  | 26824 | -0.611 | -0.3026 | Yes |
| 58 | 569184 | si:dkey-19b23.15 |  |  | 26982 | -0.628 | -0.2954 | Yes |
| 59 | 393268 | map1lc3c |  |  | 27020 | -0.631 | -0.2844 | Yes |
| 60 | 352929 | gpx4b |  |  | 27634 | -0.691 | -0.2903 | Yes |
| 61 | 100170833 | zgc:194125 |  |  | 27775 | -0.712 | -0.2810 | Yes |
| 62 | 100002893 | slc39a8 |  |  | 27777 | -0.713 | -0.2673 | Yes |
| 63 | 322425 | map1lc3b |  |  | 28058 | -0.750 | -0.2616 | Yes |
| 64 | 796322\_1 | slc3a2a |  |  | 28952 | -0.909 | -0.2721 | Yes |
| 65 | 393186 | zgc:56235 |  |  | 28953 | -0.909 | -0.2545 | Yes |
| 66 | 436651 | zgc:92066 |  |  | 29314 | -1.005 | -0.2464 | Yes |
| 67 | 100320152 | steap3 |  |  | 29967 | -1.230 | -0.2432 | Yes |
| 68 | 30590 | tp53 |  |  | 30325 | -1.401 | -0.2274 | Yes |
| 69 | 100006428 | LOC100006428 |  |  | 30579 | -1.567 | -0.2051 | Yes |
| 70 | 100126128 | zgc:173594 |  |  | 31080 | -2.058 | -0.1811 | Yes |
| 71 | 558816 | LOC558816 |  |  | 31393 | -2.629 | -0.1401 | Yes |
| 72 | 790938 | zgc:158423 |  |  | 31716 | -3.826 | -0.0764 | Yes |
| 73 | 559768 | zgc:173593 |  |  | 31762 | -4.238 | 0.0040 | Yes |
Table: GSEA details [plain text format]

  

Fig 2: FERROPTOSIS(DRE04216)      
 Blue-Pink O' Gram in the Space of the Analyzed GeneSet

  

Fig 3: FERROPTOSIS(DRE04216): Random ES distribution      
 Gene set null distribution of ES for **FERROPTOSIS(DRE04216)**
